# RETRACTED: Gu et al. Long Coding RNA XIST Contributes to Neuronal Apoptosis through the Downregulation of AKT Phosphorylation and Is Negatively Regulated by miR-494 in Rat Spinal Cord Injury. *Int. J. Mol. Sci.* 2017, *18*, 732

**DOI:** 10.3390/ijms26010347

**Published:** 2025-01-03

**Authors:** Shixin Gu, Rong Xie, Xiaodong Liu, Jiajun Shou, Wentao Gu, Xiaoming Che

**Affiliations:** Department of Neurosurgery, Huashan Hospital, Fudan University, Shanghai 200040, China; gushixin@fudan.edu.cn (S.G.); rongxiegx@126.com (R.X.); xiaodongliuxd@yeah.net (X.L.); jiajunshou@yeah.net (J.S.); wentaogu4@yeah.net (W.G.)

The journal retracts the article titled “Long Coding RNA XIST Contributes to Neuronal Apoptosis through the Downregulation of AKT Phosphorylation and Is Negatively Regulated by miR-494 in Rat Spinal Cord Injury” [1], cited above.

Following publication, concerns were brought to the attention of the Editorial Office regarding image duplication between this article [1], and an earlier publication [2], produced by a different authorship group.

Adhering to our standard procedure, the Editorial Office and Editorial Board conducted an investigation that confirmed the overlap between Figure 4A (AKT band) [1], and Figure 4F (β-actin band) [2]. While the authors collaborated within the investigation, they were unable to satisfactorily explain the overlap or provide raw material for Editorial Board evaluation. Consequently, the Editorial Board has lost confidence in the reliability of the findings and decided to retract this publication [1], as per MDPI’s retraction policy (https://www.mdpi.com/ethics#_bookmark30).

This retraction was approved by the Editor-in Chief of the *International Journal of Molecular Sciences* journal. 

The authors did not agree with this retraction.
